# Effects of* Lactobacillus gasseri* OLL2716 on* Helicobacter pylori*-Associated Dyspepsia: A Multicenter Randomized Double-Blind Controlled Trial

**DOI:** 10.1155/2016/7490452

**Published:** 2016-07-11

**Authors:** Atsushi Takagi, Hidetaka Yanagi, Hideki Ozawa, Naomi Uemura, Shigemi Nakajima, Kazuhiko Inoue, Takashi Kawai, Toshihiro Ohtsu, Yasuhiro Koga

**Affiliations:** ^1^Department of Internal Medicine, Tokai University School of Medicine, Shimokasuya, Isehara, Kanagawa 259-1193, Japan; ^2^Department of Gastroenterology, Kohnodai Hospital, National Center for Global Health and Medicine, 1-7-1 Kohnodai, Ichikawa, Chiba 272-8516, Japan; ^3^Department of Medicine, Social Insurance Shiga Hospital, 16-1 Fujimidai, Otsu, Shiga 520-0846, Japan; ^4^Department of General Medicine, Kawasaki Medical School, 577 Matsushima, Kurashiki, Okayama 701-0192, Japan; ^5^Department of Endoscopy, Tokyo Medical College, 6-7-1 Nishishijuku, Tokyo 160-0023, Japan; ^6^Research & Development Laboratories, Meiji Co. Ltd., 540 Naruta, Odawara, Kanagawa 250-0862, Japan; ^7^Division of Infectious Disease, Tokai University School of Medicine, Shimokasuya, Isehara, Kanagawa 259-1193, Japan

## Abstract

Some* Lactobacillus* spp. suppress* Helicobacter pylori* in the stomach and have potential therapeutic applications for the treatment of gastrointestinal conditions. In this study, the effects of* Lactobacillus* strains on functional dyspepsia associated with* H. pylori* infection were examined. Volunteers were screened using the ^13^C-urea breath test (UBT) and* H. pylori* stool test, and 131 participants who met the selection criteria (mean age: 48.9 years) were randomly given* L. gasseri* OLL2716-containing yogurt or placebo yogurt once daily for 12 weeks. Gastrointestinal symptoms (epigastric pain, bloating, postprandial fullness, nausea, and heartburn) and the levels of serum pepsinogen (PG), ^13^C-UBT, and* H. pylori* stool antigen were assessed. No significant differences were observed between the groups in UBT results,* H. pylori* stool antigens, or the serum PGI/II ratio. In the* L. gasseri* group, postprandial fullness was significantly lower at the end of the trial compared to the initial level (*p* < 0.05) and significantly fewer patients had a VAS score of >10 for bloating compared to the placebo group (*p* < 0.05). Dietary supplementation with* L. gasseri* OLL2716-containing yogurt may effectively suppress dyspeptic symptoms in* H. pylori*-infected patients. This study was registered at the University Hospital Medical Network Clinical Trial Registry (UMIN000016746).

## 1. Introduction

Functional dyspepsia describes a heterogeneous group of GI disorders, which may be related to atrophic gastritis. The symptoms include epigastric pain and burning and postprandial fullness, without evidence of organic disease. In Japan, functional dyspepsia has recently been recognized as a disease covered by national insurance [1]. The underlying pathophysiological mechanisms are unclear, and it has been suggested that intestinal inflammation and* H. pylori* infection may play a role [2]. The treatment of functional dyspepsia is challenging, and there is no effective therapy.

A recent pilot study indicated that a diet enriched in probiotics may alleviate dyspeptic symptoms [3]. Some strains of lactic acid bacteria, including* Lactobacillus* spp., have beneficial probiotic effects in the GI by suppressing* H. pylori* and reducing the associated inflammation [4–6]. The addition of* Lactobacillus acidophilus* to* H. pylori* treatment regimens (i.e., antibiotics) significantly improves* H. pylori* eradication rates [5, 6]. A dietary product containing* L. johnsonii* prevents* H. pylori* colonization of the GI tract in children [6], which is consistent with the results of our previous study showing that* H. pylori* cannot colonize* L. salivarius*-inoculated gnotobiotic mice [7].* Lactobacillus* spp. exhibit antimicrobial activity against* H. pylori* both in vitro and in vivo [7, 8], and beneficial effects of* Lactobacillus*-fermented milk products on* H. pylori* infection have been documented in humans [9, 10]. In a nonrandomized controlled trial, we previously found that the twice-daily consumption of yogurt containing* L. gasseri* strain OLL2716 for 8 weeks effectively treated* H. pylori* infection [9], suggesting that* H. pylori* colonization may be suppressed by the continuous ingestion of* Lactobacillus*-fermented milk products.

Despite these previous studies, the effects of dietary probiotic supplementation on functional dyspepsia are still unclear. The aims of this multicenter, double-blind, placebo-controlled clinical trial were to clarify the relationship between* H. pylori* infection and functional dyspepsia and to analyze the clinical effectiveness of* L. gasseri* OLL2716-containing yogurt consumed once daily for 12 weeks in alleviating functional dyspepsia and* H. pylori* infection.

## 2. Patients and Methods

### 2.1. Participants

The study participants were recruited from individuals who visited the hospitals (Tokai University Hospital, Social Insurance Shiga Hospital, Kawasaki Medical School Hospital, and Tokyo Medical College Hospital) for annual health checks. In the Japanese population, atrophic gastritis usually develops after the age of 30 years [11]; therefore, individuals ≥30 years old who tested positive for* H. pylori* infection were included in the study. Exclusion criteria were as follows: organic disorders, such as gastric cancer, gastric and duodenal ulcers, and pyloric stenosis; the use of nonsteroidal anti-inflammatory drugs, acid-inhibitory drugs (proton pump inhibitors or H_2_ blockers), and anti-flatulence agents; antibiotic treatment, including* H. pylori* eradication therapy, within 6 months of the study or the intention to use antibiotics for* H. pylori* eradication during the study; and the consumption of yogurt or other lactic acid bacteria-fermented beverages. A total of 131 individuals diagnosed with* H. pylori* by both the ^13^C-urea breath test (UBT; cut-off value ≥ 2.5‰) and stool antigen positivity were invited to participate and were included in the study after providing written informed consent. This clinical trial was approved by the Ethics Review Committee of each participating medical facility and was registered at the University Hospital Medical Network Clinical Trial Registry (UMIN000016746).

### 2.2. Study Protocol

The primary end-point was a decrease in* H. pylori* load assessed by the UBT and* H. pylori* antigen levels in stool samples. The secondary end-points were improvements in gastric mucosal inflammation/atrophic gastritis assessed by serum levels of pepsinogen (PG) I and pepsinogen II and changes in dyspeptic symptoms. The participants were randomly divided into two groups using a double-blind method. The placebo group (*n* = 67) received 90 g of yogurt containing milk, sugar, and stevia and fermented with* L. delbrueckii* and* Streptococcus thermophilus* (approximately 10^10^ CFU). The experimental group (*n* = 64) received the same yogurt supplemented with* L. gasseri* OLL2716 (≥10^9^ CFU). The participants were requested to consume yogurt once daily between meals for 12 weeks; the two yogurt types were identical in appearance and taste.

### 2.3. Evaluation Parameters

To assess the effects of yogurt consumption, all study parameters were measured before and after the experimental period. The participants were requested to fast on the day of the UBT. They were orally given a 100 mg UBIT tablet (Otsuka Pharmaceutical Co., Tokyo, Japan) and ^13^CO_2_ levels in the breath were measured by mass spectrometry.* H. pylori* stool antigens were detected using enzyme immunoassays (Testmate pylori antigen test; Wakamoto Pharmaceutical Co., Tokyo, Japan) and an OD value of ≥0.1 was considered positive. Serum PG levels were determined using a radioimmunoassay.

Functional dyspepsia was evaluated according to Japanese guidelines [1] using a visual analogue scale (VAS). During the study, the participants kept a diary in which they recorded compliance and scored the severity of GI symptoms (upper abdominal pain, bloating, indigestion, nausea, vomiting, and heartburn) from 1 (none) to 10 (severe pain, never experienced before). Laboratory tests (hematology, clinical chemistry, and urinalysis) were also performed before and after the completion of the study.

During the product consumption phase, 3 and 4 participants from the experimental and placebo groups, respectively, used prohibited concomitant drugs and were excluded from the analysis. Therefore, data were compared between 61* L. gasseri* OLL2716 yogurt consumers and 63 placebo yogurt consumers (*n* = 124 in total).

### 2.4. Statistical Analysis

The number of patients required to detect a difference of 0.5% between the groups was calculated. Based on a decrease in the expected mean of 0.5%, a significance level of 5%, and a power of 80%, at least 102 subjects in total were required. Participants' characteristics were compared between the groups using Pearson's *χ* ^2^ test. Stool antigen, UBT, PG, and VAS results before and after product consumption were compared using the Wilcoxon signed rank sum test. The Mann-Whitney test was used for comparisons between groups. The data are expressed as medians (range), and the level of statistical significance was set at *p* < 0.05. All statistical analyses were performed using SPSS 11.5J Windows software (SPSS Japan Inc., Tokyo, Japan).

## 3. Results and Discussion

There were no significant differences in the demographic and initial clinical characteristics (Table 1) or compliance between the* L. gasseri* and placebo groups, and none of the participants experienced any adverse events during the study.

**Figure 1:**
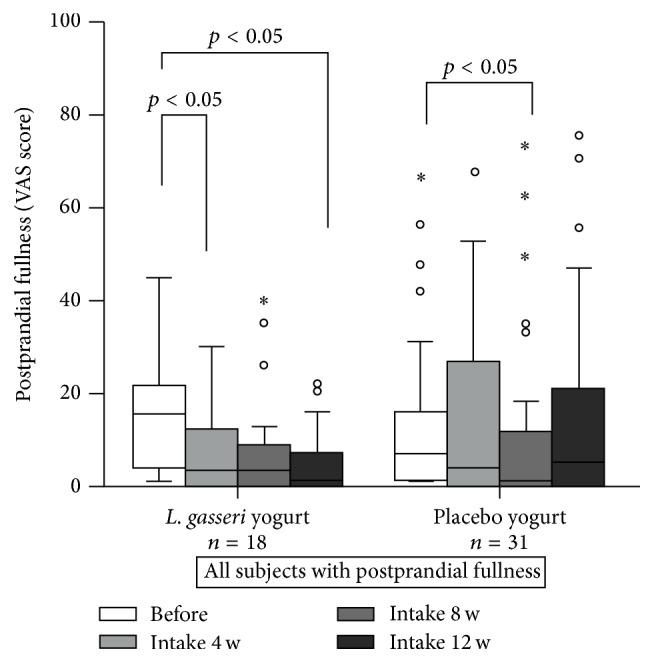
Changes in the severity score for postprandial fullness. Participants were asked to take* Lactobacillus gasseri* OLL2716-containing yogurt or placebo yogurt (control) once daily for 12 weeks, and the severity of dyspeptic symptoms was analyzed according to a visual analogue scale (VAS) as follows: 1, no symptoms; 10, severe pain, never experienced before. ∘: less than 3 times the height of the box. *∗*: more than 1.5 times the height of the box.

**Figure 2:**
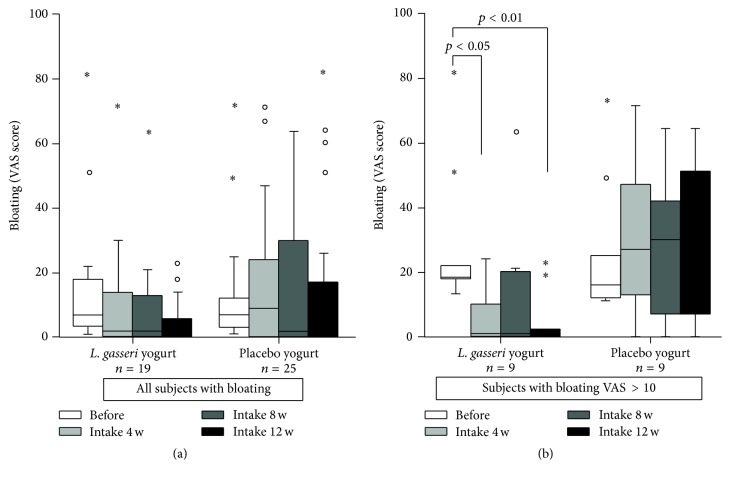
Changes in the bloating severity score. Participants were asked to take* Lactobacillus gasseri* OLL2716-containing yogurt or placebo yogurt (control) once daily for 12 weeks and the severity of dyspeptic symptoms was analyzed according to a visual analogue scale (VAS) as follows: 1, no symptoms; 10, severe pain, never experienced before. (a) The total number of participants with bloating; (b) the number of participants with a bloating severity score of >10. ∘: less than 3 times the height of the box. *∗*: more than 1.5 times the height of the box.

There were no significant differences in the UBT or stool* H. pylori* antigen values between the start and end of the study in either group (Table 2). Serum PGI and PGII levels in both groups were significantly higher at the end of the study period compared to the initial levels (*p* < 0.05), and the PGI/II ratios remained unchanged (Table 2). Based on the VAS results, the incidences of gastrointestinal symptoms before the test products were consumed were as follows: epigastric pain, 23.4%; bloating, 35.5%; postprandial fullness, 39.5%; nausea, 28.2%; and heartburn, 29.8% (Table 3). At least one of these symptoms was reported by 58% of the participants (72 out of 124), and these patients were considered dyspeptic. The VAS score for postprandial fullness in the* L. gasseri* group was significantly higher before than after the consumption of the test yogurt (*p* < 0.05; Figure 1). Although no significant differences were observed in bloating according to the VAS analysis, the number of subjects with a VAS bloating score of >10 was significantly lower in the* L. gasseri* group than in the placebo group (*p* < 0.05; Figure 2). Overall, these results indicate that the consumption of* L. gasseri* OLL2716-containing yogurt alleviated some dyspeptic symptoms in individuals infected with* H. pylori*.

In our previous study, we found that the consumption of* L. gasseri* OLL2716-containing yogurt twice daily for 8 weeks reduced the density of* H. pylori* and ameliorated gastritis in 31 patients, as evidenced by a significant decrease in UBT values and increase in the PGI/II ratio [9]. Furthermore, it improved* H. pylori* eradication rates when it was included as a first-line triple therapy with antibiotics [5]. In this study, we tested once-daily yogurt intake to promote compliance to the study protocol over a longer experimental period (12 weeks). This intake schedule did not produce significant changes in the UBT and stool antigen results or the PGI/II ratio. In other words, the reduced dose used in this study was not sufficient to eradicate* H. pylori*.

PG levels are associated with gastric mucosa functional activity, and a PGI/PGII ratio of <3 is a marker of atrophic gastritis [12]. In patients with* H. pylori* infection, increases in serum PGII concentrations to greater than 12 ng/mL and decreases in the PGI/PGII ratio to below 4.0 were used as the cut-offs for the diagnosis of* H. pylori* infection, with sensitivity and specificity of 90.0% and 93.5%, respectively [13]. However, the effects of probiotics on serum PG levels are unclear. Igarashi et al. [14] have shown that the ingestion of* L. gasseri* increases PGI levels in proton pump inhibitor users, whereas Miki et al. [15] have reported that fermented milk containing* Bifidobacterium bifidum* decreases PGI and the PGI/II ratio in patients with mild mucosal atrophy. The mechanisms by which* L. gasseri* and other probiotic bacteria influence PG levels remain unknown and require further investigation.

Functional dyspepsia presents a major economic burden in modern society. Despite the high cost of investigating and treating functional dyspepsia, few therapeutic options are currently available. Systematic reviews have suggested that prokinetic therapy and the suppression of acid release via proton pump inhibitors may alleviate dyspeptic symptoms [16, 17]. However, an effective treatment for functional dyspepsia has yet to be established.

Although* H. pylori* infection induces changes in gastric emptying, gastrointestinal motility, gastric acid secretion, and the perception of gastric characteristics related to functional dyspepsia, the role of* H. pylori* in the pathogenesis of dyspepsia remains controversial [2, 18]. Thus, it is still unclear whether the eradication of* H. pylori* is beneficial for functional dyspepsia, and clinical trials have not revealed a clear association between* H. pylori* eradication and the relief of dyspeptic symptoms [2, 19].

Probiotics have been suggested to treat functional dyspepsia, but studies examining their effects are inconclusive. For example, a previous study of the effects of probiotic-enriched olive oil on dyspeptic symptoms in 44 individuals revealed a significant amelioration of nausea, pain/discomfort in the abdomen, and postprandial fullness compared to the control group [3]. However, it was a proof-of-concept study and had a very short duration (i.e., 1 week).

The current multicenter double-blind, randomized, placebo-controlled study had a duration of 12 weeks. We showed that the VAS scores for postprandial fullness and the number of patients with a bloating score of >10 decreased significantly after the consumption of* L. gasseri*-containing yogurt (*p* < 0.05), indicating long-term benefits of the probiotic for the relief of dyspeptic symptoms.* L. gasseri* OLL2716 has multiple beneficial properties, including acid resistance and adhesion to gastric epithelial cells in vitro (unpublished data), as well as successful competition with* H. pylori* for colonization of the GI tract [9]. Our data further suggest that dietary supplementation with this* Lactobacillus* strain has potential applications for the treatment of functional dyspepsia.

Our study had some limitations. First, the sample size of* H. pylori*-infected individuals with functional dyspepsia was small because the participants were recruited from people admitted to the hospitals for an annual health check. However, a power analysis indicated that this sample size was sufficient to detect differences at the 5% level. Second, we did not account for life-style habits, such as smoking and alcohol consumption, which can have confounding effects. In our future studies, we plan to address this issue. Finally, we did not strictly adhere to the Rome III criteria for functional dyspepsia [20]. Almost all Japanese citizens are covered by health insurance and can seek medical help within a month of the onset of dyspeptic symptoms; thus, these patients do not meet the Rome III criterion of a symptom duration of 6 months. We used our own questionnaire, rather than a standard questionnaire, as recommended by the Japanese guidelines for functional dyspepsia, such as the Gastrointestinal Symptom Rating Scale (GSRS). Further studies on functional dyspepsia according to the Rome III criteria are necessary to elucidate the role of probiotics, including* L. gasseri* OLL2716, on dyspeptic symptoms.

## 4. Conclusions

We demonstrated that a low dose of the* L. gasseri* strain OLL2716 improves dyspeptic symptoms, without inhibiting* H. pylori* infection. Further studies that examine higher probiotic doses and larger patient cohorts using standard diagnostic criteria for functional dyspepsia are needed to evaluate the effects of* L. gasseri* OLL2716 on the relief of dyspeptic symptoms.

## Figures and Tables

**Table 1:** Participant characteristics.

	*Lactobacillus gasseri* group (*n* = 61)		Placebo group (*n* = 63)		Group comparison^*∗*^
	Median	25–75 percentile	Median	25–75 percentile	*p* value
Age	51.0	44.0–56.0	48	41.0–54.0	0.214
Gender (male/female)	27/34		17/46		0.06
Stool Hp antigens	1.45	0.38–2.38	1.217	0.38–2.83	0.622
UBT	19.4	10.4–35.0	19.6	10.3–36.7	0.924
PGI/PGII	2.6	2.1–3.5	2.7	2.0–3.9	0.468
PGI	61.0	45.2–76.5	59.2	42.0–74.5	0.589
PGII	21.4	16.4–32.5	20.6	16.9–27.5	0.635

^*∗*^Mann-Whitney test.

**Table 2:** Effects of *Lactobacillus gasseri *consumption on *Helicobacter pylori *infection and serum pepsinogen levels.

Parameters	Group	Before		After		Before versus after	Group comparison
		Median	25–75 percentile	Median	25–75 percentile	*p* value^*∗*^	*p* value^*∗∗*^
Stool Hp antigen	*L. gasseri* group	1.45	0.38–2.38	1.17	0.48–2.13	0.588	0.958
	Placebo group	1.22	0.38–2.83	1.24	0.44–2.50	0.584	

UBT	*L. gasseri* group	19.4	10.4–35.0	21.9	11.1–31.2	0.395	0.516
	Placebo group	19.6	10.3–36.7	19.9	10.3–32.8	0.958	

PGI/PGII	*L. gasseri* group	2.6	2.1–3.5	2.7	2.1–3.6	0.537	0.414
	Placebo group	2.7	2.0–3.9	2.7	2.1–3.7	0.604	

PGI	*L. gasseri* group	61.0	45.2–76.5	63.3	48.5–79.6	0.006	0.497
	Placebo group	59.2	42.0–74.5	62.3	43.9–77.0	0.018	

PGII	*L. gasseri* group	21.4	16.4–32.5	22.5	16.1–35.1	0.012	0.725
	Placebo group	20.6	16.9–27.5	24.3	16.4–29.8	0.003	

*L. gasseri* group, *n* = 61; placebo group, *n* = 63.

^*∗*^Wilcoxon signed rank sum test.

^*∗∗*^Mann-Whitney test.

**Table 3:** Dyspeptic symptoms.

Symptom	*Lactobacillus gasseri* group (*n* = 61)			Placebo group (*n* = 63)		
	Before, *n* (%)	After, *n* (%)	*p*	Before, *n* (%)	After, *n* (%)	*p*
Postprandial fullness	18/61 (29.5%)^*∗*^	20/61 (32.8%)	0.696	31/63 (49.2%)	24/61 (38.1%)	0.209
Epigastric pain	8/61 (13.1%)^*∗∗*^	13/61 (21.3%)	0.230	21/63 (33.3%)	27/63 (42.9%)	0.271
Heartburn	17/61 (27.9%)	17/61 (27.9%)	1.000	20/63 (31.7%)	20/63 (31.7%)	1.000
Nausea	16/61 (26.2%)	15/61 (24.6%)	0.835	19/63 (30.1%)	17/63 (27.0%)	0.693
Bloating	19/61 (31.1%)	21/61 (34.4%)	0.700	25/63 (39.7%)	29/63 (23.8%)	0.471

^*∗*^ *p* = 0.025 and ^*∗∗*^ *p* = 0.008 versus the placebo group.
